# Feasibility of electronic patient-reported outcome monitoring and self-management program in aplastic anemia and paroxysmal nocturnal hemoglobinuria—a pilot study (ePRO-AA-PNH)

**DOI:** 10.1007/s00277-022-05012-5

**Published:** 2022-11-03

**Authors:** Silas Bänziger, Kimmo Weisshaar, Reetta Arokoski, Sabine Gerull, Jörg Halter, Alicia Rovó, Mario Bargetzi, Jeroen S. Goede, Yuliya Senft, Sabine Valenta, Jakob R. Passweg, Beatrice Drexler

**Affiliations:** 1 Division of Hematology, University Hospital Basel, Petersgraben 4, 4031 Basel, Switzerland; 2 Kaiku Health Ltd, Helsinki, Finland; 3 Department of Hematology and Central Hematology Laboratory, Inselspital, Bern University Hospital, Bern, Switzerland; 4 Division of Hematology, University Medical Clinic, Kantonsspital Aarau AG, Aarau, Switzerland; 5 Division of Oncology and Haematology, Kantonsspital Winterthur, Winterthur, Switzerland; 6 Nursing Science, Department Public Health, University of Basel, Basel, Switzerland

**Keywords:** ePRO, Patient-reported outcomes, Electronic health record, Self-management, Aplastic anemia, Paroxysmal nocturnal hemoglobinuria

## Abstract

**Introduction:**

Electronic patient-reported outcomes (ePRO) are increasingly recognized in health care, as they have been demonstrated to improve patient outcomes in cancer, but have been less studied in rare hematological diseases. The aim of this study was to develop and test the feasibility of an ePRO system specifically customized for aplastic anemia (AA) and paroxysmal nocturnal hemoglobinuria (PNH).

**Methods:**

After performing a user-centered design evaluation an ePRO system for AA and PNH patients could be customized and the application was tested by patients and their medical teams for 6 months. Symptom-reporting triggered self-management advice for patients and prompts them to contact clinicians in case of severe symptoms, while the medical team received alerts of severe symptoms for patient care.

**Results:**

All nine included patients showed a high adherence rate to the weekly symptom-reporting (72%) and reported high satisfaction. The system was rated high for usage, comprehensibility, and integration into daily life. Most patients (78%) would continue and all would recommend the application to other AA/PNH patients. Technical performance was rarely a barrier and healthcare providers saw ePRO-AA-PNH as a useful supplement, but the lacking integration into the hospital information system was identified as a major barrier to usage.

**Conclusion:**

An ePRO system customized for AA and PNH was feasible in terms of adherence, satisfaction, and performance, showing a high potential for these rare conditions in terms of data collection and patient guidance. However, the integration into clinical workflows is crucial for further routine use.

**Trial registration:**

ClinicalTrials.gov NCT04128943.

**Supplementary Information:**

The online version contains supplementary material available at 10.1007/s00277-022-05012-5.

## Introduction

Patient-reported outcomes (PRO) are defined as any report of the status of a patient’s health condition that comes directly from the patient, without interpretation by a clinician. Electronically gathered PRO (ePRO) additionally provides real-time notifications to health care providers on alarming symptoms and has shown high levels of compliance [1–6]. In cancer patients, this approach has repeatedly shown to enhance patient-clinician communication and to facilitate rapid clinical intervention, resulting in less emergency visits, improved overall survival, and health-related quality of life (HR-QoL) [7–9].

Based on this extensive experience of ePRO systems in cancer, aplastic anemia (AA) and paroxysmal nocturnal hemoglobinuria (PNH) patients are ideal candidates for such ePRO interventions. AA and PNH are rare chronic conditions, characterized by symptoms of bone marrow failure such as fatigue, bleeding, and infections [10–12] in AA and by complications of hemolysis and thrombosis in PNH [13, 14]. Despite major improvements in therapies in the past decades, AA and PNH patients often remain symptomatic with significantly reduced HR-QoL as well as requiring a long-term follow-up due to their risk for severe complications and late effects [15–19].

In this setting, symptom monitoring with ePROs may have the potential to detect symptoms and life-threatening complications earlier, thereby improving disease management. In these rare entities, ePROs may also help to guide patients remotely when living a greater distance from their specialized medical center. To date, the ePRO system has neither been adapted to the needs of this patient group nor been tested in AA/PNH patients.

The aim of this pilot study was to develop a disease-specific ePRO system for routine symptom monitoring with self-management advice and warning system, which later was examined for feasibility in AA and/or PNH patients.

## Methods

### Study design and study population

This single-center study used qualitative and quantitative methods to test the feasibility of an ePRO system adapted to the needs of AA and PNH patients. Between November and December 2019, nine patients with AA and/or PNH and their corresponding medical team were prospectively enrolled in the study at the University Hospital Basel. Patients were eligible if they were older than 18 years and had Internet access with prior email experience. Patients with mental alteration or psychiatric disease, which would compromise informed consent or study adherence, as well as patients, who were not able to read or write German were excluded. Participation in the study was offered by the treating physician, and after the patient’s approval to participate, the patients were approached by the study team by telephone, email, or during routine visits for study inclusion. The study was approved by the local ethics committee (EKNZ Nr. 2019–01,563) and registered under ClinicalTrials.gov (NCT04128943). All parts of the study were performed within the principles of the Declaration of Helsinki.

### Disease-specific ePRO system customization

The ePRO system provided by Kaiku Health® (Helsinki, Finland) was utilized for symptom monitoring. This browser-based platform has been used in cancer patients before [20, 21] and is accessible by computers, tablets, and smartphones with access to the Internet.

For this study, the system was adapted from its original form to the specific needs of AA and PNH patients using feedback from patients, nurses, and physicians [22, 23]. We primarily included two disease-specific symptom questionnaires, each assessing 11 core symptoms in AA/PNH, which we previously had developed by scoping review, consensus rounds with patients and medical experts, and the adaption of questions of the patient-reported outcome version of the common terminology criteria for adverse events (PRO-CTCAE) [24]. At study inclusion and study end, the EORTC-QLQ-C30 questionnaire was used for assessing HR-QoL. An overview of the application development is shown in Fig. 2, and further questionnaire and interview detail in the supplemental material are shown in Tables 3 and 4.

**Fig. 1:**
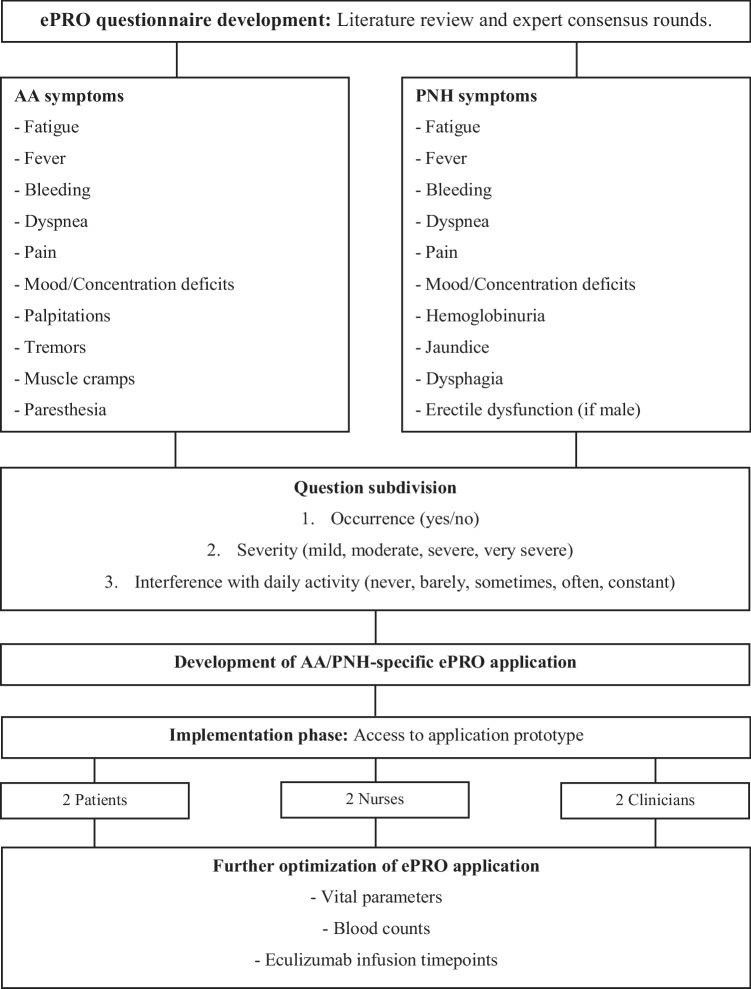
Application development. Steps of the application development until the feasibility study

### Clinical test phase

After giving informed consent, patients were instructed by the study investigator on how to use the application. All patients were assigned to a symptom monitoring program depending on their diagnosis and sex (AA, PNH female, PNH male), which consisted of a weekly symptom questionnaire and an email reminder. Previous work has identified an optimal recall time of 7 days [25]. In addition to the questionnaire, the patients were asked to measure and document their vital parameters weekly (blood pressure, pulse, and temperature) and to fill out a HR-QoL questionnaire in the first and last study week. Optionally, they could report their current blood counts and if applicable the timepoint of eculizumab infusion within the application.

The ePRO system incorporated a predefined algorithm to stratify patient-reported symptoms in real-time into minor, moderate, and severe symptoms [24], each resulting in different actions (Tables 1 and 4, Fig. 4): In case of minor or moderate symptoms, patients would automatically receive self-management advice. Severe symptoms would generate emergency first aid instructions and a warning to consult their medical team or, if not available, the emergency room.

**Table 1:** Symptom management by the ePRO application

Symptom grade	ePRO system action	Example of ePRO feedback for fatigue
Grade 0: No symptoms	No action required	No action
Grade 1: Mild symptoms	Self-management advice for patients	“What you can do: - Stay active – physically and mentally! - Moderate physical activity (a.e. swimming, walking, cycling, dancing) can have a positive impact for your energy level *- and more (see Table 4, Supplemental Material)*
Grade 2: Moderate symptoms	(I) Self-management advice for patients (II) Notification for the medical team, without actions taken	As above
Grade 3: Severe symptoms	(I) Alert for patients to contact their medical team (II) First Aid instructions for patients (III) Alert to the medical team	You have indicated severe symptoms, which should be evaluated. Please contact your care-team (evening or night: medical officer in charge or emergency room)

Two dedicated physicians of the research team reviewed patient responses within the application daily and reported severe symptoms to the responsible physicians at the treatment center. All involved physicians and nurses were invited to use the application for their daily routine work. It was left to the responsible physician on how to further manage the care of the patient with respect to reported symptoms.

Participants tested the application for a total of 6 months. During this period, patient interviews by telephone were conducted at 3 weeks and at 3 months. Upon study completion, patients as well as the involved nurses and physicians evaluated the application in a final interview.

### Data analysis

In this feasibility study, we assessed the recruitment, adherence, user experience, and technical performance of an ePRO system. PRO questionnaire adherence rates were quantified by calculating the proportions of completed weekly questionnaires compared to the observation period in weeks. Based on recent implementation guidelines [22], we conducted semi-structured interviews for the assessment of usability, efficiency, user satisfaction, and technical issues of the application and evaluated the results using thematic analysis. The frequency and severity of patient-reported symptoms were summarized descriptively. Quantitative statistical analyses were performed using R version 4.01 (R core team, Vienna, AT).

## Results

### Patient characteristics and recruitment

During November and December 2019, 14 patients were screened and asked to participate in the study. Four patients did not want to participate due to a lack of motivation (*n* = 4, 29%, median age: 27 years IQR 26–30), and one patient was excluded due to limited German language skills (*n* = 1, 7%). Nine patients were enrolled, resulting in a recruitment rate of 64%. The median age was 35 years (IQR 29–56), whereby more females (*n* = 6, 67%) than males (*n* = 3, 33%) were included in the study. Five patients were diagnosed with AA, three with PNH, and one with overlapping AA/PNH. The median disease duration was 9 years (IQR 6–13). Further patient characteristics are listed in Table 2.

**Table 2:** Patient characteristics

	Pt 1	Pt 2	Pt 3	Pt 4	Pt 5	Pt 6	Pt 7	Pt 8	Pt 9
Age	56	22	19	60	65	35	29	50	34
Sex	F	F	M	F	M	M	F	F	F
Disease	PNH	MAA	SAA	AA	SAA	SAA	PNH	SAA-PNH^1^	PNH
Disease duration	9 years	6 years	7 months	37 years	6 years	10 years	2 years	19 years	13 years
Remission status in AA/treatment response in PNH at inclusion^2^	Partial response	No treatment since diagnosis	Partial response	Partial response	Relapse	Relapse	Major response	Complete response (PNH clone 78%)	Partial response
Previous treatments for AA/PNH	-	-	ATG/CSA/Eltrombopag	3 × ALG, CSA	CSA mono, Eltrombopag, ATG/CSA	ATG/CSA Eltrombopag	-	ATG/CSA	-
Treatment during study period for AA/PNH	Eculizumab (900 mg every 14 days)	Watch & Wait	CSA/Eltrombopag	Ec transfusions due to residual PRCA	Allogeneic HSCT from MUD at 3 months	Allogeneic HSCT from haploidentical donor at 3 months	Eculizumab (900 mg every 14 days)	CSA	Eculizumab (900 mg every 14 days)
QoL^3^ Start	83	83	83	83	50	50	83	100	67
QoL^3^ End	83	67	-	83	58	67	-	100	67

*CSA* Ciclosporin A, *Ec* erythrocyte concentrate, *HSCT* hematopoietic stem cell transplantation, *MUD* matched–unrelated donor, *ATG* anti-thymocyte-globulin, *ALG* antilymphocyte globulin, *PRCA* pure red cell aplasia

^1^Assigned to PNH follow-up

^2^Remission status in AA according to NIH criteria/treatment response in PNH according to Severe Aplastic Anemia Working Party (SAAWP) criteria

^3^QoL: EORTC QLQ-C30 score (version 3.0)

### Patient adherence

A total of 234 weekly reminders were sent and 168 questionnaires were completed, resulting in a 72% questionnaire adherence rate. Figure 3 illustrates the monthly adherence rate, showing a decreasing rate from 91% (month 1) to 53% (month 6) over the total study period. Besides the weekly questionnaires, an additional questionnaire was filled out in 12 instances.

**Fig. 2:**
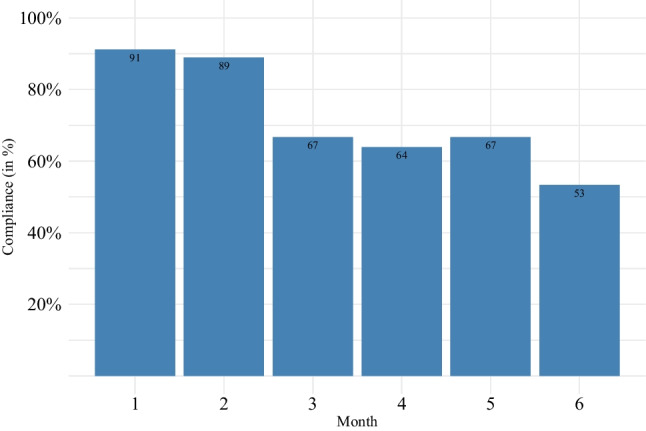
Patient adherence to the weekly questionnaire over the 6-month study time

### Symptom reporting and triggered actions

Figure 1 demonstrates all reported symptoms for AA and PNH, respectively. A total of 331 symptoms were reported, of which 154 (46.5%) were classified as mild, 95 (28.7%) as moderate, and 82 (24.8%) as severe. The most common reported symptom was fatigue in AA (44 entries) and in PNH (43 entries). Symptoms of severe grade were most often reported as bleeding in AA (14 entries) and fatigue in PNH (21 entries). Eighty-two symptoms were graded as severe according to the predefined algorithm, 28 due to symptom severity, 23 due to interference with daily activity, and 31 due to both reasons equally. Each symptom per patient over time is shown in Table 6 in the supplemental material: five patients reported symptoms during the whole study period with increasing and decreasing or stable severity; three patients stopped reporting symptoms after 13, 18, and 23 weeks, respectively; and one patient stopped reporting symptoms from weeks 8–16 in between the study period.

**Fig. 3:**
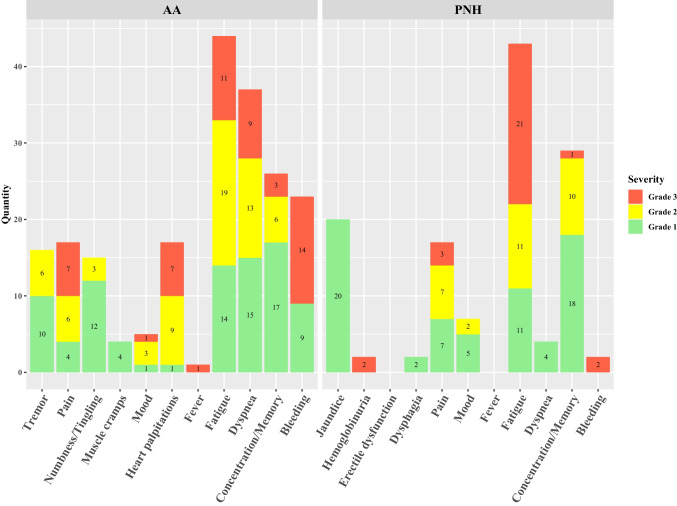
Reported symptoms. Grade 1 corresponds to mild and grade 2 to moderate symptoms, which both resulted in self-management advice for the patients, and grade 3 corresponds to severe symptoms, sending out additionally an alert to the patients and physicians

Severe symptoms led to 36 alerts being sent out to the treating physicians. These alerts did not lead to additional physician contacts or admissions of the patients. In addition to the predefined symptom questions, additional symptoms reported were nausea, cough, itching, and chest pressure.

Besides symptom monitoring, vital parameters were documented 81 times by 6 patients, resulting in an overall adherence of 35%. Blood values were documented by 5 patients with a total of 41 entries. The timepoint of eculizumab infusion was documented by one of the three PNH patients receiving eculizumab.

### User experience and technical performance

All patients, two nurses, and five physicians reported on their experience with the ePRO application. Technical problems were reported in 3 cases, all of which were due to difficulties with a password reset (two nurses and one physician). Patients did not report any technical problems or concerns on data security. The application was mostly accessed by smartphone (*n* = 7, 78%). Overall, the usability of the application was rated as “easy to use” while the symptom questionnaire was classified as “easy to understand” by all patients.

One patient mentioned difficulties in grading the severity of symptoms. Of the five patients who reported severe symptoms, two stated that the warnings and recommendations to contact a physician were in line with their personal experience and symptom management. Three patients reported that the warnings were only sometimes in agreement with their personal experience, of which all three stated that the warning for “fatigue” was triggered too soon. Four patients never triggered an automatic warning.

Reasons for non-adherence were forgetting (*n* = 4, 44%), being too busy (*n* = 4, 44%), the lack of any symptoms (*n* = 1, 11%), and not wanting to be reminded of the disease (*n* = 1, 11%). While some patients (*n* = 3, 33%) deemed the self-instructions for symptom care useful, almost half of the patients (*n* = 4, 44%) regarded the self-instructions as “not very useful” since they were already trained in disease management for many years (median disease duration 9 years). They suggested that newly diagnosed patients would benefit the most from this application. All patients rated the time expenditure as “appropriate.” While 6 patients (67%) would like to continue to use the app weekly, 2 patients (22%) would prefer to use it less often (every 2 weeks), and 1 patient (11%) more often (every 3 days). The integration of the app into daily life was rated as “very easy” by all patients.

Overall, all patients reported being satisfied with the application. While 7 patients (78%) would continue to use the app, 2 patients (22%) would stop using it. All patients would recommend the application to other AA or PNH patients. Recommendations for improvement focused on the automatic integration of vital parameters, blood values, and medication into the app, a calendar function for medication and hospital visits, a possibility to ask the care-team questions, and voluntary social functions.

All members of the medical team described the application as “easy to use.” While more than half of the medical team members (*n* = 4, 57%) reported a benefit from the tool, others (*n* = 3, 43%) did not. Both nurses felt confident in their ability to screen and manage the reported symptoms. Though not everybody saw a personal benefit from the tool, all caretakers would continue to use the application due to the likely benefit provided to patients. All recommended that the tool should be integrated into the electronic hospital records and clinical work flows. To improve data interpretation, it was suggested to integrate data on therapies into the application. Another suggestion was an alarm function for patients directly integrated into the start page of the application. Data extraction from the interviews can be found in the supplemental materials (Tables 7, 8, and 9).

## Discussion

This pilot study confirmed that electronic symptom monitoring including a warning system is feasible for both patients and their clinicians in AA and PNH. To our knowledge, this is the first study that assessed an ePRO system in AA and PNH, as previous ePRO trials predominantly focussed on cancer patients [1, 7, 8].

Patient interest and recruitment rate for the ePRO system were high, suggesting that this approach can be used broadly for these rare conditions. Overall, patients’ adherence to the weekly symptom questionnaire was also high (72%), but decreased over time with the highest rates at the beginning (91%) and the lowest rates after 6 months (53%). Severe symptoms (e.g., during allogeneic stem cell transplantation) seemed to be a barrier, whereas technical issues were rarely a hurdle to use the tool. Patients reported as the main reason for reduced adherence that they forgot to report symptoms, which is in line to other ePRO studies [1, 5]. We also observed that patients with minor occasional or without symptoms stopped using the tool prematurely. This study was performed as an add-on to routine workflows, so neither medical staff nor patients saw it as part of their core disease management. Consequently, in order to become part of routine care and increase compliance, it is pivotal for ePROs to overcome these organizational barriers by integrating them into clinical workflows and hospital information systems [23, 26]. In this aspect, responsibilities, time resources, and management during non-office hours of the medical team have to be defined for practice usability [27, 28]. This also holds true for the collection of vital parameters, blood counts, and therapies, which were documented infrequently in our study and underlines the need for a direct interface with the hospital information system for automatic synchronization, emphasizing that successful implementation can only be achieved by allocating enough resources to IT integration and ongoing support.

In contrast to previous studies in cancer patients [7, 8], the warning system did not result in any additional hospital admissions. Since it is a key aim of ePRO systems to guide patients to contact their medical team on time before symptoms worsen and cause complications, we further investigated the reasons for this finding. Some patients stated that they managed symptoms already on their own or called their physicians before filling out the ePRO questionnaire, reflecting the long disease history of many of the included patients (median: 9 years) and indicating that the tool might have a bigger impact on AA/PNH patients early in their disease journey. The ePRO system resulted in different responses and recall ratios in this patient group as previously reported in cancer patients (e.g., more frequent psychosocial issues such as fatigue and concentration problems rather than worrisome bleeding, dyspnea, or dysphagia), and this tool might have less impact on hard endpoints such as overall survival or complication rate, which however has to be assessed in a larger cohort as our study was only a feasibility study. We still believe that digital health tools could have a meaningful clinical impact in AA/PNH, possibly more on HR-QoL issues, which have high importance for patients. These tools will be even more relevant in the future when more patients are treated as outpatients with less control due to upcoming new complement inhibitors administered at home or improved oral medications for AA (e.g. thrombopoietin agonists). In the long term, such tools could guide patients through their whole disease journey including all aspects of medical care (medication, vaccination, appointments, laboratory results, and chart information). However, automatic data transfer/security and incorporation into hospital information systems is again pivotal for successful implementation.

Another core result of our study was the difficulty to assess certain symptoms adequately (e.g., fatigue), which might have led to underreporting [12]. This reveals a major drawback of short ePRO questionnaires (10–12 items), which can be completed quickly (max. 15 min), but miss out on details needed to assess complex symptoms. This highlights that it might be of additional value to refine instruments for certain symptoms in AA and PNH such as “fatigue”—as one of the most common symptoms often persisting despite adequate therapy—or “neurocognitive deficits.” Unfortunately, such specific instruments for assessing these symptoms in AA/PNH are not yet available, although for cancer-related fatigue [29] already well established. Interestingly, the tool could detect less known symptoms (e.g., concentration problems) for this disease entity, which could point towards an underestimation of these symptoms in AA/PNH. However, higher patient numbers are needed to reliably assess the symptom burden in AA/PNH with the help of ePRO and the reliability of the used questionnaire.

Overall, the user experience was very positive. Most patients and clinicians would continue to use the application and the medical team regarded the tool as a useful addition to routine management. In particular, for inexperienced physicians, the tool might be especially helpful to estimate the severity and monitor the disease course, as it is difficult to gain extensive clinical experience in these rare diseases. The multidimensional recording of symptoms, blood values, and therapies could also form the basis for an automatic prediction of adverse events and disease courses as has been attempted in other fields [30, 31], in particular when considering the future benefits of artificial intelligence and machine learning.

With the focus on the rare occurrence of AA and PNH, several needs of this ePRO system for the future could be identified: A calendar function with reminders for questionnaire completion, medication intake, and hospital visits may also improve drug and ePRO compliance [32, 33], which increasingly gets important as emerging therapies in this field can be administered at home. Also, a social function could provide patients with a valuable opportunity to create a network and improve patient engagement while far away from their specialized medical centers. Considering the current COVID-19 pandemic, this tool is also of great value to directly communicate with the care team in a convenient and remote way as recently shown successful in cancer patients [34]. Nevertheless, such electronic tools might bear risks for patients, as they could underreport symptoms or handle mild symptoms falsely by the self-management recommendations. It is therefore pivotal that the tool includes short-term reminders for re-evaluation of the symptom severity and advice to contact their medical team. Ultimately, patients still should be seen by medical experts regularly. Besides the utility in patient care, this disease-specific ePRO system might be useful in collecting data for future trials seeking real-world information on patient symptom burden and quality of life.

Several limitations of the study merit consideration. First, the small patient sample does not allow generalization and may have overestimated adherence rates. However, disease prevalence rates for our region were met and patient engagement and feedback were high. Studies with a higher number of patients, particularly newly diagnosed patients and/or patients receiving novel treatments in the field, are needed to assess the definite clinical impact of ePRO for AA and PNH.

## Conclusion

It was feasible to customize a disease-specific ePRO system for AA/PNH patients and their medical team, showing a high potential for these rare and chronic conditions in terms of adherence, satisfaction, and performance. However, the integration into clinical workflows is crucial for routine use and the clinical benefit has yet to be assessed by more patients. This ePRO system can form the basis for further usage in AA/PNH patients, enabling the collection of real-world data within trials in this rare disease, but also guiding patients in their disease journey.

## Supplementary Information

Below is the link to the electronic supplementary material.

Supplementary file1 (DOC 718 KB)

## Data Availability

The datasets generated during and/or analyzed during the current study are available from the corresponding author on reasonable request.
