# Supplementary material for: Feasibility of electronic patient-reported outcome monitoring and self-management program in aplastic anemia and paroxysmal nocturnal hemoglobinuria—a pilot study (ePRO-AA-PNH)
Source: Ann Hematol. 2022 Nov 3;102(1):199–208. doi: 10.1007/s00277-022-05012-5 (PMC9631592; doi:10.1007/s00277-022-05012-5)
Supplement: Supplementary file 1 — Supplementary file1 (DOC 718 KB) [file 277_2022_5012_MOESM1_ESM.doc]

**Table 3. List of final questionnaire items and source**

| **Questionnaire item** | **Source** |
| --- | --- |
| Fatigue | PRO-CTCAE Symptom Term: Fatigue |
| Fever | Center specific diagnostic and treatment guidelines based on Petersdorf et al. (1) |
| Bleeding | Modified WHO Bleeding Scale (2) |
| Dyspnea | PRO-CTCAE Symptom Term: Shortness of breath |
| Pain | According to PRO-CTCAE Symptom Term: General pain |
| Mood | According to PRO-CTCAE Symptom Term: Sad; and based on an instrument by Whooley et al. (3) |
| Concentration/memory | Combination of PRO-CTCAE Symptom Term: Concentration and PRO-CTCAE Symptom Term: Memory |
| Open question on other symptom | N/A |
| Items only for AA questionnaire: |  |
| Palpitations | PRO-CTCAE Symptom Term: Heart palpitations |
| Tremor | According to CTCAE V5.0 Term: Tremor and adopted to PRO-CTCAE style |
| Muscle cramps | According to CTCAE V5.0 Term: Muscle cramp and adopted to PRO-CTCAE style. |
| Paresthesia, numbness | PRO-CTCAE™ Symptom Term: Numbness & tingling |
| Items only for PNH questionnaire: |  |
| Hemoglobinuria | N/A |
| Jaundice | N/A |
| Dysphagia | According to CTCAE V5.0 Term: Dysphagia and adopted to the PRO-CTCAE Symptom Term: Difficulty swallowing |
| Erectile dysfunction | PRO-CTCAE Symptom Term: Achieve and maintain erection |

WHO: World Health Organization, PRO-CTCAE: patient-reported outcome version of the Common Terminology Criteria for Adverse Events, N/A: not applicable

1. Petersdorf RG, Beeson PB. Fever of unexplained origin: report on 100 cases. Medicine. 1961;40(1):1-30

2. Kaufman RM, Djulbegovic B, Gernsheimer T, Kleinman S, Tinmouth AT, Capocelli KE, et al. Platelet transfusion: a clinical practice guideline from the AABB. Ann Intern Med. 2015;162(3):205-13.

3. Whooley MA, Avins AL, Miranda J, Browner WS. Case‐finding instruments for depression: Two questions are as good as many. J Gen Intern Med. 1997;12(7):439-45

**Table 4: Interviews for user-centered redesign**

|  | Patient 1 (AA) | Patient 2 (PNH) | Nurse 1 | Nurse 2 |
| --- | --- | --- | --- | --- |
| How would you rate your computer experience? | Basic knowledge | Very good | Very good |  |
| How would a tool like this help? | Instructions for nutrition and hygiene, informations about GvHD and skin lesions. | Advice from physicians and other patients to cope with the symptoms. | Improvement for self-evaluation and patient empowerment. |  |
| In which situation would you use the tool? | During free time |  |  |  |
| What should the tool contain? | Tips for daily life. | General information about disease and therapy, blood values. | Medical data and advice for patients, symptom management, support with medication | Calendar function (with treatment days and reminder for vaccination). Traffic light system for mild-moderate-severe. |
| When would you use the tool? | Times of symptom worsening. |  | Once weekly for patients, nurses depending on time expenditure |  |
| Login: | Easy |  |  |  |
| Navigation: | Easy | Easy | Easy |  |
| Overview: | Good overview | Good to have | Well-structured and similar for patients and nurses |  |
| User interface: | Clear | Good design. | Clear |  |
| Potential time expenditure: | 5-10 minutes per week | 30 minutes per week | <30 minutes per week |  |
| Device: | Smartphone | Smartphone |  |  |
| Which information should be included? | Skin lesions, nausea, signs for hemolysis. | Daily life informations (job, symptoms, medication), new developments | Symptom management (infection, bleeding, fatigue) without the need for a consultation. | Contact information of physicians and patient support group. |
| Suggestions for improvement: | Questionnaires for each therapy and gender. | Network possibility between patients | Mobile version. Hospital should be able to enter blood values. | Patient-friendly and simple interface. |
| Lacking items: | Remind-function for medication. | Reminder for vaccinations, eculizumab documentation. | Documentation of blood values. Help with medication and stress situations. | Documentation of blood values. |
| How would the tool helped you with your disease? | Help with symptoms and when to contact the medical team. | With general information on how to cope with the disease. |  |  |

**Table 5: ePRO feedback**

| Symptom (Severity) | Application feedback |
| --- | --- |
| Fatigue (mild, moderate) | - Stay active – physically and mentally! - Moderate physical activity (e.g. Swimming, walking, cycling, dancing, gymnastics) can have a positive effect on your energy levels. - Keep a regular and sufficient sleep rhythm. Don’t make long power naps, longer inactivity can worsen your fatigue! - Plan your days ahead and take into account the times you know to be more or less energetic. Set priorities and reserve your energy for things of importance. - Change in between phases of activity and rest. Rest before and after exhausting activities. |
| Fatigue (severe) | You have indicated severe symptoms, which should be evaluated. Please contact your care-team (evening or night: medical officer in charge or emergency room). |
| Fever | Fever should always be evaluated. Please contact your care-team (evening or night: medical officer in charge or emergency room).  The following measures can be taken at home, these do not replace contact with your careteam:   - Go easy on yourself. - Drink enough fluids. - Against fever you may take 1000mg Paracetamol (max. every 6 hours), if no hypersensitivity is known - If you have low white blood cells and you were prescribed emergency antibiotics (e.g. Tavanic®), please take these as prescribed |
| Nose bleeding (mild) | 1. Blow your nose once (this can worsen the bleeding for a short time, which is okay).  2. Sit or stay while leaning slightly forward with the upper body (do not lay down your head backwards!)  3. Press both sides of the nose under the bony part of the nose and keep the nose pressed shut for 15-20 minutes. Do not test in between, if the nose is still bleeding.  4. If the bleeding hasn’t stopped, keep the nose shut for another 15 minutes.  5. If the bleedings stops, avoid blowing your nose or manipulation with the nose.  If the nose bleeding still continues, contact your care-team! |
| Nose bleeding (severe) | Severe nose bleeding should always be evaluated. Please contact your care-team (evening or night: medical officer in charge or emergency room).  The following measures can be taken at home, these do not replace contact with your careteam:   - See nose bleeding (mild) |
| Mouth bleeding (mild) | - If you were prescribed Cyklokapron®, dissolve it into 20ml of water and use half of it as rinse. Keep the rinse in the mouth for 2-3 minutes, after that spit it out or swallow it. - Gum bleeding can be a sign of dental disease. Follow a strong mouth hygiene (link in knowledge section). |
| Mouth bleeding (severe) | You have indicated severe symptoms, which should be evaluated. Please contact your care-team (evening or night: medical officer in charge or emergency room). |
| Skin and soft tissue bleeding (mild) | - Bruises resolve by themselves. - To ease bruises, you can cool them (for 15 minutes every 2 hours) and elevate the affected body part. - We advise against superficial heparin gel because of its blood thinning effect. - For light pain, Paracetamol (1g, max. 4x/day) can help. For strong pain we advise to see a physician. |
| Skin and soft tissue bleeding (severe) | You have indicated severe symptoms, which should be evaluated. Please contact your care-team (evening or night: medical officer in charge or emergency room). |
| Gastrointestinal bleeding (mild) | - Keep to the advice of the physician which has detected the faecal occult blood. |
| Gastrointestinal bleeding (severe) | Fresh blood in stool or vomit, tarry stools or dark brown vomit always needs to be evaluated. Please contact your care-team (evening or night: medical officer in charge or emergency room). |
| Blood in urine (mild) | - Keep to the advice of the physician which has detected the blood in urine. |
| Blood in urine (severe) | Blood colours the urine to red-brown. Blood shouldn’t be there and needs to be evaluated. Please contact your care-team (evening or night: medical officer in charge or emergency room). |
| Vaginal bleeding (pregnant) | - Inform your physician about the pregnancy. - Vaginal bleeding during pregnancy always needs to be evaluated. Please contact your care-team (evening or night: medical officer in charge or emergency room). |
| Vaginal bleeding (not pregnant) | Monthly menstrual bleedings are normal for women. Contact your care-team if:   - Menstrual bleeding is very strong (≥4 tampons within 2 hours or blood clots) - Menstruation lasts longer than 8 days. - You’re pregnant. - It’s not a menstrual bleeding, but a breakthrough bleeding or bleeding after menopause |
| Blood coughing | Coughing of bloodstained secretion or bigger amounts of blood always needs to be evaluated. Please contact your care-team (evening or night: medical officer in charge or emergency room). |
| Dyspnoea (mild, moderate) | The following measures can aid, although they do not replace contact with the care-team if dyspnoea is severe:   - Keep calm and sit. - Cool and moving air can improve dyspnoea. Stay in cool areas and use a fan. - Fear can worsen dyspnoea, therefore breathing and relaxation exercises can help. |
| Dyspnoea (severe) | You have indicated severe symptoms, which should be evaluated. Please contact your care-team (evening or night: medical officer in charge or emergency room). |
| Pain (mild, moderate) | Pain can occur in different places, levels and durations and the treatment depends on these factors. Contact your care-team if the pain is very strong, new, continuous or strange to you.  In a first step you can:   - Take pain medication, if prescribed by your physician - Locally use warming or cooling patches. - Try relaxation exercises (music, breathing exercises, meditation) and distraction - Try to stay as active as possible, this can ease pain - Try gentle massages |
| Pain (severe) | You have indicated severe symptoms, which should be evaluated. Please contact your care-team (evening or night: medical officer in charge or emergency room). |
| Mood (mild, moderate) | - Rise rapidly in the morning - Try to stay physically fit and add regular physical activity to your daily life. Physical exercises can help to improve mood, reduce stress and improve concentration and sleep. - Keep a persistent daily structure. - Talk to somebody close. - Share your thoughts with your physician and get professional help. |
| Mood (severe) | You have indicated severe symptoms, which should be evaluated. Please contact your care-team (evening or night: medical officer in charge or emergency room). |
| Concentration/memory problems (mild, moderate) | The following measures can help with concentration and memory:   - Regular physical activity - Balanced diet - Consistent sleep pattern with enough sleep - Social activities - Keeping the brain active (learn new things, read, solve riddles) |
| Concentration/memory problems (severe) | You have indicated severe symptoms, which should be evaluated. Please contact your care-team (evening or night: medical officer in charge or emergency room). |
| Palpitations (mild, moderate) | Contact your care-team, if:   - You feel an irregular heart beat - Palpitations continue for more than 30 minutes - You experience more than one fit per week   These measures can help prevent palpitations, but do not replace contact with your care-team if the points mentioned above apply:   - Reduce caffeine, nicotine and alcohol consume - Avoid stress situations, relaxation exercises may help - Regular physical activity - Drink enough water - Weight reduction (if overweight) - Optimal control of blood pressure |
| Palpitations (severe) | You have indicated severe symptoms, which should be evaluated. Please contact your care-team (evening or night: medical officer in charge or emergency room). |
| Tremor (mild, moderate) | - Control your blood pressure: if it’s too high or low (systolic <100mmHg or >160mmHg, respectively) contact your care-team. In case of low blood pressure, fluid intake may help as well as leg elevation. - A disruption of body salt levels may worsen your symptoms. If you were prescribed magnesium by your physician, you may take it according to the prescription. - Uncontrollable trembling may be a side effect of Ciclosporin or Tacrolimus. Discuss at your next physician visit the possibility of a dose reduction. |
| Tremor (severe) | You have indicated severe symptoms, which should be evaluated. Please contact your care-team (evening or night: medical officer in charge or emergency room). |
| Muscle cramps (mild, moderate) | - If you were prescribed magnesium by your physician, you may take it according to the prescription. - Do stretching exercises regularly - Wear shoes with good support - Drink enough water and reduce alcohol and coffee intake - Having acute cramps, you can try the following:   - Stretch the cramped muscle   - Walk around and shake the cramped extremity   - Lie down and elevate the cramped extremity   - Shower the cramped extremity with warm water for 5 minutes or take a warm bath   - Rub the cramped muscle with freezer packages |
| Muscle cramps (severe) | You have indicated severe symptoms, which should be evaluated. Please contact your care-team (evening or night: medical officer in charge or emergency room). |
| Numbness/tingling (mild, moderate) | - Try to keep the affected parts warm (e.g. hand warmers, hot beverages, warm baths) - Avoid the cold, this might worsen the sensation disturbance - Rubbing and gentle massages may help - Regular physical activities (e.g. cycling, swimming, running) may help to reduce the symptoms - If prescribed by your physician, pain medication may help temporary |
| Numbness/tingling (severe) | You have indicated severe symptoms, which should be evaluated. Please contact your care-team (evening or night: medical officer in charge or emergency room). |
| Dark urine: Hemoglobinuria in PNH patients | You have indicated severe symptoms, which should be evaluated. Please contact your care-team (evening or night: medical officer in charge or emergency room). |
| Jaundice (mild, moderate) | This can be a sign of hemolysis. If the symptom is new to you, tell your physician soon. If the symptom is known, note the recommendations from your care-team. |
| Jaundice (severe) | You have indicated severe symptoms, which should be evaluated. Please contact your care-team (evening or night: medical officer in charge or emergency room). |
| Dysphagia (mild, moderate) | - Solid food should be cooked soft or if necessary pureed. - Pay attention to drink enough during the meal - Cold and acidic food seen to support the swallowing reflex - Keep an upright position while eating - If choking repeatedly, you can try to bend the head forwards during eating - If you choke while drinking, you may use thickening agents after consulting your physician |
| Dysphagia (severe) | You have indicated severe symptoms, which should be evaluated. Please contact your care-team (evening or night: medical officer in charge or emergency room). |
| Erectile dysfunction | There are many reasons for erectile dysfunction and a lot of treatment options. Speak with your hematological specialist and perhaps with an urologist about this problem!  If an erection lasts longer than 2 hours, contact your care-team immediately (evening or night: medical officer in charge or emergency room)! |


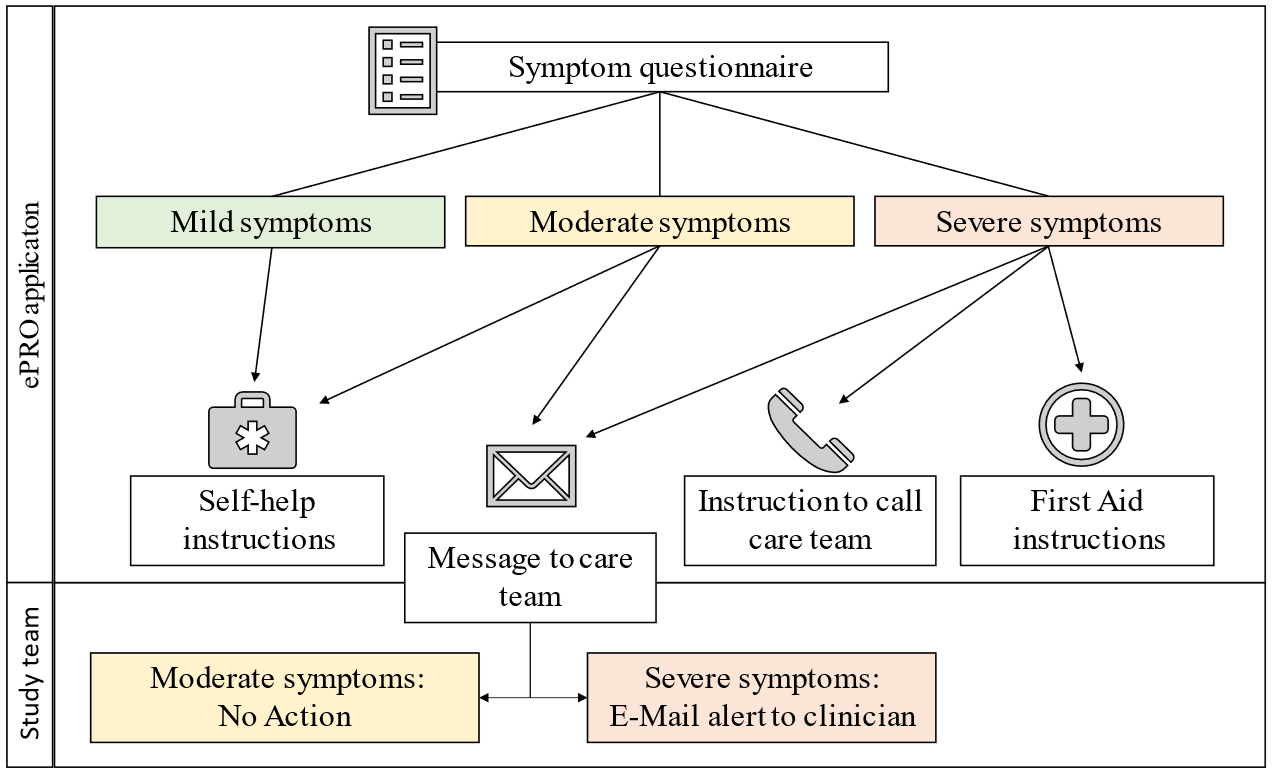
**Figure 4: ePRO actions**

**Table 6: Symptoms reported for each patient per week**

Patient 1, female, PNH

| week | 1 | 2 | 3 | 4 | 5 | 6 | 7 | 8 | 9 | 10 | 11 | 12 | 13 | 14 | 15 | 16 | 17 | 18 | 19 | 20 | 21 | 22 | 23 | 24 | 25 | 26 |
| --- | --- | --- | --- | --- | --- | --- | --- | --- | --- | --- | --- | --- | --- | --- | --- | --- | --- | --- | --- | --- | --- | --- | --- | --- | --- | --- |
| Bleeding | 3 | 0 | 0 | 0 | 0 | 0 | 0 | 0 |  | 0 | 0 | 0 | 0 | 0 |  | 0 | 0 | 0 | 0 | 0 |  | 0 |  |  | 0 | 0 |
| Fever | 0 | 0 | 0 | 0 | 0 | 0 | 0 | 0 |  | 0 | 0 | 0 | 0 | 0 |  | 0 | 0 | 0 | 0 | 0 |  | 0 |  |  | 0 | 0 |
| Dyspnoea | 1 | 0 | 0 | 1 | 0 | 0 | 1 | 0 |  | 1 | 0 | 0 | 0 | 0 |  | 0 | 0 | 0 | 0 | 0 |  | 0 |  |  | 0 | 0 |
| Fatigue | 2 | 0 | 0 | 1 | 1 | 1 | 1 | 2 |  | 3 | 1 | 0 | 3 | 0 |  | 0 | 0 | 0 | 3 | 1 |  | 0 |  |  | 0 | 0 |
| Concentration / memory problems | 0 | 0 | 0 | 0 | 0 | 0 | 0 | 1 |  | 1 | 0 | 0 | 0 | 1 |  | 1 | 0 | 0 | 0 | 0 |  | 0 |  |  | 0 | 0 |
| Pain | 1 | 0 | 0 | 1 | 0 | 0 | 0 | 0 |  | 0 | 0 | 0 | 2 | 0 |  | 0 | 0 | 0 | 0 | 0 |  | 0 |  |  | 0 | 0 |
| Mood | 0 | 0 | 0 | 0 | 1 | 0 | 0 | 2 |  | 0 | 1 | 1 | 0 | 2 |  | 1 | 0 | 0 | 0 | 1 |  | 0 |  |  | 0 | 0 |
| Jaundice | 1 | 1 | 1 | 1 | 1 | 1 | 1 | 1 |  | 1 | 1 | 0 | 1 | 1 |  | 1 | 1 | 1 | 1 | 1 |  | 1 |  |  | 1 | 1 |
| Dysphagia | 0 | 0 | 0 | 0 | 0 | 0 | 0 | 1 |  | 0 | 0 | 0 | 0 | 0 |  | 0 | 0 | 0 | 0 | 0 |  | 0 |  |  | 0 | 0 |
| Hemoglobinuria | 0 | 0 | 0 | 0 | 0 | 0 | 0 | 3 |  | 0 | 0 | 0 | 3 | 0 |  | 0 | 0 | 0 | 0 | 0 |  | 0 |  |  | 0 | 0 |
| QLQ-30 | 83.3 |  |  |  |  |  |  |  |  |  |  |  |  |  |  |  |  |  |  |  |  |  |  |  |  | 83.3 |

Patient 2, female, AA

| week | 1 | 2 | 3 | 4 | 5 | 6 | 7 | 8 | 9 | 10 | 11 | 12 | 13 | 14 | 15 | 16 | 17 | 18 | 19 | 20 | 21 | 22 | 23 | 24 | 25 | 26 |
| --- | --- | --- | --- | --- | --- | --- | --- | --- | --- | --- | --- | --- | --- | --- | --- | --- | --- | --- | --- | --- | --- | --- | --- | --- | --- | --- |
| Bleeding | 0 | 0 | 0 | 0 |  | 0 | 0 |  | 0 | 0 | 0 | 0 | 0 |  |  |  |  |  |  |  |  |  |  |  |  |  |
| Fever | 0 | 0 | 0 | 0 |  | 0 | 0 |  | 0 | 0 | 0 | 0 | 0 |  |  |  |  |  |  |  |  |  |  |  |  |  |
| Dyspnoea | 0 | 0 | 0 | 0 |  | 0 | 0 |  | 0 | 0 | 0 | 0 | 0 |  |  |  |  |  |  |  |  |  |  |  |  |  |
| Muscle cramps | 0 | 0 | 0 | 0 |  | 0 | 0 |  | 0 | 0 | 1 | 0 | 0 |  |  |  |  |  |  |  |  |  |  |  |  |  |
| Fatigue | 0 | 0 | 0 | 0 |  | 0 | 0 |  | 0 | 0 | 0 | 0 | 0 |  |  |  |  |  |  |  |  |  |  |  |  |  |
| Palpitations | 0 | 0 | 0 | 0 |  | 0 | 0 |  | 0 | 0 | 0 | 0 | 0 |  |  |  |  |  |  |  |  |  |  |  |  |  |
| Concentration / memory problems | 0 | 0 | 0 | 0 |  | 0 | 0 |  | 0 | 0 | 0 | 0 | 0 |  |  |  |  |  |  |  |  |  |  |  |  |  |
| Pain | 0 | 0 | 0 | 0 |  | 0 | 0 |  | 0 | 0 | 2 | 0 | 0 |  |  |  |  |  |  |  |  |  |  |  |  |  |
| Mood | 0 | 0 | 0 | 0 |  | 0 | 0 |  | 0 | 0 | 0 | 0 | 0 |  |  |  |  |  |  |  |  |  |  |  |  |  |
| Numbness / tingling | 0 | 0 | 0 | 0 |  | 0 | 0 |  | 0 | 0 | 0 | 0 | 0 |  |  |  |  |  |  |  |  |  |  |  |  |  |
| Tremor | 0 | 0 | 0 | 0 |  | 0 | 0 |  | 0 | 0 | 0 | 0 | 0 |  |  |  |  |  |  |  |  |  |  |  |  |  |
| QLQ-30 | 83 |  |  |  |  |  |  |  |  |  |  |  |  |  |  |  |  |  |  |  |  |  |  |  |  | 67 |

Patient 3, male, AA

| week | 1 | 2 | 3 | 4 | 5 | 6 | 7 | 8 | 9 | 10 | 11 | 12 | 13 | 14 | 15 | 16 | 17 | 18 | 19 | 20 | 21 | 22 | 23 | 24 | 25 | 26 |
| --- | --- | --- | --- | --- | --- | --- | --- | --- | --- | --- | --- | --- | --- | --- | --- | --- | --- | --- | --- | --- | --- | --- | --- | --- | --- | --- |
| Bleeding | 0 | 0 | 0 | 1 | 0 | 0 | 0 | 0 | 0 | 0 |  | 0 | 0 |  | 1 | 0 | 0 |  | 0 | 0 |  | 0 |  |  |  |  |
| Fever | 0 | 0 | 0 | 0 | 0 | 0 | 0 | 0 | 0 | 0 |  | 0 | 0 |  | 0 | 0 | 0 |  | 0 | 0 |  | 0 |  |  |  |  |
| Dyspnoea | 1 | 0 | 0 | 0 | 0 | 0 | 0 | 0 | 0 | 0 |  | 1 | 0 |  | 0 | 0 | 0 |  | 0 | 0 |  | 0 |  |  |  |  |
| Muscle cramps | 0 | 0 | 0 | 0 | 0 | 0 | 0 | 0 | 1 | 1 |  | 0 | 0 |  | 0 | 0 | 0 |  | 0 | 0 |  | 0 |  |  |  |  |
| Fatigue | 1 | 1 | 1 | 0 | 0 | 0 | 0 | 2 | 1 | 1 |  | 0 | 0 |  | 0 | 0 | 0 |  | 0 | 0 |  | 0 |  |  |  |  |
| Palpitations | 0 | 0 | 0 | 0 | 0 | 0 | 0 | 0 | 0 | 0 |  | 0 | 0 |  | 0 | 0 | 0 |  | 0 | 0 |  | 0 |  |  |  |  |
| Concentration / memory problems | 2 | 2 | 1 | 1 | 1 | 1 | 1 | 1 | 1 | 0 |  | 0 | 0 |  | 0 | 1 | 0 |  | 0 | 0 |  | 0 |  |  |  |  |
| Pain | 0 | 0 | 0 | 0 | 0 | 0 | 0 | 0 | 0 | 0 |  | 0 | 0 |  | 0 | 0 | 0 |  | 0 | 0 |  | 0 |  |  |  |  |
| Mood | 0 | 0 | 2 | 1 | 0 | 0 | 0 | 0 | 0 | 0 |  | 0 | 0 |  | 2 | 0 | 2 |  | 0 | 0 |  | 0 |  |  |  |  |
| Numbness / tingling | 2 | 2 | 2 | 1 | 1 | 1 | 1 | 1 | 1 | 1 |  | 1 | 1 |  | 1 | 1 | 0 |  | 0 | 0 |  | 0 |  |  |  |  |
| Tremor | 0 | 1 | 0 | 0 | 0 | 0 | 0 | 0 | 0 | 0 |  | 0 | 0 |  | 0 | 0 | 0 |  | 0 | 0 |  | 0 |  |  |  |  |
| QLQ-30 | 83 |  |  |  |  |  |  |  |  |  |  |  |  |  |  |  |  |  |  |  |  |  |  |  |  |  |

Patient 4, female, AA

| week | 1 | 2 | 3 | 4 | 5 | 6 | 7 | 8 | 9 | 10 | 11 | 12 | 13 | 14 | 15 | 16 | 17 | 18 | 19 | 20 | 21 | 22 | 23 | 24 | 25 | 26 |
| --- | --- | --- | --- | --- | --- | --- | --- | --- | --- | --- | --- | --- | --- | --- | --- | --- | --- | --- | --- | --- | --- | --- | --- | --- | --- | --- |
| Bleeding | 0 | 0 | 0 | 0 | 0 | 0 | 0 | 0 | 0 | 0 | 0 | 0 |  | 0 | 0 | 0 | 0 | 0 | 0 | 0 | 0 | 0 | 0 | 0 | 0 | 0 |
| Fever | 0 | 0 | 0 | 0 | 0 | 0 | 0 | 0 | 0 | 0 | 0 | 0 |  | 0 | 0 | 0 | 0 | 0 | 0 | 0 | 0 | 0 | 0 | 0 | 0 | 0 |
| Dyspnoea | 0 | 0 | 0 | 0 | 0 | 0 | 0 | 0 | 0 | 0 | 0 | 0 |  | 0 | 0 | 0 | 0 | 0 | 0 | 0 | 0 | 0 | 0 | 0 | 0 | 0 |
| Muscle cramps | 0 | 0 | 0 | 0 | 0 | 0 | 0 | 0 | 0 | 0 | 0 | 0 |  | 0 | 0 | 0 | 0 | 0 | 0 | 0 | 0 | 0 | 0 | 0 | 0 | 0 |
| Fatigue | 0 | 3 | 0 | 0 | 0 | 0 | 0 | 0 | 0 | 0 | 0 | 0 |  | 2 | 0 | 0 | 0 | 0 | 0 | 0 | 0 | 0 | 0 | 0 | 0 | 0 |
| Palpitations | 0 | 0 | 0 | 0 | 0 | 0 | 0 | 0 | 0 | 0 | 0 | 0 |  | 3 | 0 | 0 | 0 | 0 | 0 | 0 | 0 | 0 | 0 | 0 | 0 | 0 |
| Concentration / memory problems | 0 | 0 | 0 | 0 | 0 | 0 | 0 | 0 | 0 | 0 | 0 | 0 |  | 0 | 0 | 0 | 0 | 0 | 0 | 0 | 0 | 0 | 0 | 0 | 0 | 0 |
| Pain | 2 | 3 | 0 | 0 | 0 | 0 | 0 | 3 | 0 | 0 | 0 | 0 |  | 3 | 0 | 0 | 0 | 0 | 0 | 0 | 0 | 0 | 0 | 0 | 0 | 0 |
| Mood | 0 | 0 | 0 | 0 | 0 | 0 | 0 | 0 | 0 | 0 | 0 | 0 |  | 3 | 0 | 0 | 0 | 0 | 0 | 0 | 0 | 0 | 0 | 0 | 0 | 0 |
| Numbness / tingling | 0 | 0 | 0 | 0 | 0 | 0 | 0 | 0 | 0 | 0 | 0 | 0 |  | 0 | 0 | 0 | 0 | 0 | 0 | 0 | 0 | 0 | 0 | 0 | 0 | 0 |
| Tremor | 0 | 0 | 0 | 0 | 0 | 0 | 0 | 0 | 0 | 0 | 0 | 0 |  | 0 | 0 | 0 | 0 | 0 | 0 | 0 | 0 | 0 | 0 | 0 | 0 | 0 |
| QLQ-30 | 83 |  |  |  |  |  |  |  |  |  |  |  |  |  |  |  |  |  |  |  |  |  |  |  |  | 83 |

Patient 5, male, AA

| week | 1 | 2 | 3 | 4 | 5 | 6 | 7 | 8 | 9 | 10 | 11 | 12 | 13 | 14 | 15 | 16 | 17 | 18 | 19 | 20 | 21 | 22 | 23 | 24 | 25 | 26 |
| --- | --- | --- | --- | --- | --- | --- | --- | --- | --- | --- | --- | --- | --- | --- | --- | --- | --- | --- | --- | --- | --- | --- | --- | --- | --- | --- |
| Bleeding | 3 | 0 | 0 | 1 | 3 | 3 | 3 | 3 | 3 |  | 3 | 3 | 3 |  | 3 | 3 | 1 | 0 | 0 |  | 0 | 0 |  | 0 |  | 0 |
| Fever | 0 | 0 | 0 | 0 | 0 | 0 | 0 | 0 | 0 |  | 0 | 0 | 0 |  | 3 | 0 | 0 | 0 | 0 |  | 0 | 0 |  | 0 |  | 0 |
| Dyspnoea | 1 | 2 | 2 | 3 | 2 | 2 | 1 | 2 | 3 |  | 3 | 0 | 2 |  | 0 | 0 | 0 | 2 | 3 |  | 2 | 1 |  | 1 |  | 1 |
| Muscle cramps | 0 | 0 | 0 | 0 | 1 | 0 | 0 | 0 | 0 |  | 0 | 0 | 0 |  | 0 | 0 | 0 | 0 | 0 |  | 0 | 0 |  | 0 |  | 0 |
| Fatigue | 2 | 2 | 2 | 3 | 3 | 2 | 2 | 3 | 2 |  | 2 | 2 | 2 |  | 2 | 1 | 1 | 3 | 3 |  | 2 | 2 |  | 1 |  | 1 |
| Palpitations | 3 | 2 | 2 | 2 | 2 | 3 | 3 | 3 | 3 |  | 0 | 0 | 0 |  | 0 | 3 | 0 | 0 | 0 |  | 0 | 0 |  | 0 |  | 0 |
| Concentration / memory problems | 0 | 0 | 0 | 0 | 0 | 0 | 0 | 0 | 0 |  | 0 | 0 | 0 |  | 0 | 0 | 0 | 0 | 0 |  | 0 | 0 |  | 0 |  | 0 |
| Pain | 0 | 0 | 0 | 3 | 0 | 0 | 0 | 0 | 0 |  | 0 | 0 | 0 |  | 0 | 0 | 0 | 0 | 0 |  | 0 | 0 |  | 0 |  | 2 |
| Mood | 0 | 0 | 0 | 0 | 0 | 0 | 0 | 0 | 0 |  | 0 | 0 | 0 |  | 0 | 0 | 0 | 0 | 0 |  | 0 | 0 |  | 0 |  | 0 |
| Numbness / tingling | 0 | 0 | 0 | 0 | 0 | 0 | 0 | 0 | 0 |  | 0 | 0 | 0 |  | 0 | 0 | 0 | 0 | 0 |  | 0 | 0 |  | 0 |  | 0 |
| Tremor | 0 | 0 | 0 | 0 | 0 | 0 | 0 | 0 | 0 |  | 0 | 0 | 0 |  | 0 | 0 | 0 | 0 | 0 |  | 0 | 0 |  | 0 |  | 0 |
| QLQ-30 | 50 |  |  |  |  |  |  |  |  |  |  |  |  |  |  |  |  |  |  |  |  |  |  |  |  | 58.3 |

Patient 6, male, AA

| week | 1 | 2 | 3 | 4 | 5 | 6 | 7 | 8 | 9 | 10 | 11 | 12 | 13 | 14 | 15 | 16 | 17 | 18 | 19 | 20 | 21 | 22 | 23 | 24 | 25 | 26 |
| --- | --- | --- | --- | --- | --- | --- | --- | --- | --- | --- | --- | --- | --- | --- | --- | --- | --- | --- | --- | --- | --- | --- | --- | --- | --- | --- |
| Bleeding | 0 | 0 |  | 0 |  | 1 |  | 1 |  |  |  |  |  |  |  |  | 0 | 0 | 0 | 0 | 0 | 0 | 0 | 0 | 0 | 0 |
| Fever | 0 | 0 |  | 0 |  | 0 |  | 0 |  |  |  |  |  |  |  |  | 0 | 0 | 0 | 0 | 0 | 0 | 0 | 0 | 0 | 0 |
| Dyspnoea | 3 | 3 |  | 3 |  | 3 |  | 3 |  |  |  |  |  |  |  |  | 2 | 1 | 1 | 1 | 1 | 1 | 1 | 2 | 2 | 2 |
| Muscle cramps | 0 | 0 |  | 0 |  | 0 |  | 0 |  |  |  |  |  |  |  |  | 0 | 0 | 0 | 0 | 0 | 0 | 0 | 0 | 0 | 0 |
| Fatigue | 3 | 3 |  | 3 |  | 3 |  | 3 |  |  |  |  |  |  |  |  | 1 | 0 | 0 | 0 | 0 | 1 | 2 | 1 | 2 | 2 |
| Palpitations | 2 | 2 |  | 2 |  | 2 |  | 2 |  |  |  |  |  |  |  |  | 1 | 0 | 0 | 0 | 0 | 0 | 0 | 0 | 0 | 0 |
| Concentration / memory problems | 3 | 2 |  | 3 |  | 3 |  | 2 |  |  |  |  |  |  |  |  | 1 | 0 | 1 | 1 | 1 | 1 | 1 | 1 | 2 | 2 |
| Pain | 0 | 0 |  | 0 |  | 0 |  | 0 |  |  |  |  |  |  |  |  | 0 | 2 | 0 | 0 | 0 | 0 | 0 | 0 | 0 | 0 |
| Mood | 0 | 0 |  | 0 |  | 0 |  | 0 |  |  |  |  |  |  |  |  | 0 | 0 | 0 | 0 | 0 | 0 | 0 | 0 | 0 | 0 |
| Numbness / tingling | 0 | 0 |  | 0 |  | 1 |  | 0 |  |  |  |  |  |  |  |  | 0 | 0 | 0 | 0 | 0 | 0 | 0 | 0 | 0 | 0 |
| Tremor | 1 | 0 |  | 0 |  | 1 |  | 1 |  |  |  |  |  |  |  |  | 2 | 2 | 1 | 1 | 2 | 2 | 1 | 1 | 1 | 2 |
| QLQ-30 | 50 |  |  |  |  |  |  |  |  |  |  |  |  |  |  |  |  |  |  |  |  |  |  |  |  | 67 |

Patient 7, female, PNH

| week | 1 | 2 | 3 | 4 | 5 | 6 | 7 | 8 | 9 | 10 | 11 | 12 | 13 | 14 | 15 | 16 | 17 | 18 | 19 | 20 | 21 | 22 | 23 | 24 | 25 | 26 |
| --- | --- | --- | --- | --- | --- | --- | --- | --- | --- | --- | --- | --- | --- | --- | --- | --- | --- | --- | --- | --- | --- | --- | --- | --- | --- | --- |
| Bleeding | 0 |  | 0 | 0 | 0 | 0 | 0 | 0 | 0 |  |  | 0 |  |  |  |  |  | 0 |  |  |  |  |  |  |  |  |
| Fever | 0 |  | 0 | 0 | 0 | 0 | 0 | 0 | 0 |  |  | 0 |  |  |  |  |  | 0 |  |  |  |  |  |  |  |  |
| Dyspnoea | 0 |  | 0 | 0 | 0 | 0 | 0 | 0 | 0 |  |  | 0 |  |  |  |  |  | 0 |  |  |  |  |  |  |  |  |
| Fatigue | 0 |  | 1 | 0 | 0 | 0 | 0 | 0 | 0 |  |  | 0 |  |  |  |  |  | 0 |  |  |  |  |  |  |  |  |
| Concentration / memory problems | 0 |  | 0 | 0 | 0 | 0 | 0 | 0 | 0 |  |  | 0 |  |  |  |  |  | 0 |  |  |  |  |  |  |  |  |
| Pain | 0 |  | 0 | 0 | 0 | 0 | 0 | 0 | 0 |  |  | 0 |  |  |  |  |  | 0 |  |  |  |  |  |  |  |  |
| Mood | 0 |  | 0 | 0 | 0 | 0 | 0 | 0 | 0 |  |  | 0 |  |  |  |  |  | 0 |  |  |  |  |  |  |  |  |
| Jaundice | 0 |  | 0 | 0 | 0 | 0 | 0 | 0 | 0 |  |  | 0 |  |  |  |  |  | 0 |  |  |  |  |  |  |  |  |
| Dysphagia | 0 |  | 0 | 0 | 0 | 0 | 0 | 0 | 0 |  |  | 0 |  |  |  |  |  | 0 |  |  |  |  |  |  |  |  |
| Hemoglobinuria | 0 |  | 0 | 0 | 0 | 0 | 0 | 0 | 0 |  |  | 0 |  |  |  |  |  | 0 |  |  |  |  |  |  |  |  |
| QLQ-30 | 83 |  |  |  |  |  |  |  |  |  |  |  |  |  |  |  |  |  |  |  |  |  |  |  |  |  |

Patient 8, female, AA-PNH

| week | 1 | 2 | 3 | 4 | 5 | 6 | 7 | 8 | 9 | 10 | 11 | 12 | 13 | 14 | 15 | 16 | 17 | 18 | 19 | 20 | 21 | 22 | 23 | 24 | 25 | 26 |
| --- | --- | --- | --- | --- | --- | --- | --- | --- | --- | --- | --- | --- | --- | --- | --- | --- | --- | --- | --- | --- | --- | --- | --- | --- | --- | --- |
| Bleeding | 0 | 0 | 0 | 0 | 0 | 0 | 0 | 0 | 0 | 0 | 0 | 0 | 0 | 0 | 0 | 0 | 0 | 0 | 0 | 0 | 0 | 0 | 0 | 0 | 0 | 0 |
| Fever | 0 | 0 | 0 | 0 | 0 | 0 | 0 | 0 | 0 | 0 | 0 | 0 | 0 | 0 | 0 | 0 | 0 | 0 | 0 | 0 | 0 | 0 | 0 | 0 | 0 | 0 |
| Dyspnoea | 0 | 0 | 0 | 0 | 0 | 0 | 0 | 0 | 0 | 0 | 0 | 0 | 0 | 0 | 0 | 0 | 0 | 0 | 0 | 0 | 0 | 0 | 0 | 0 | 0 | 0 |
| Fatigue | 0 | 0 | 0 | 0 | 0 | 0 | 0 | 0 | 0 | 0 | 0 | 0 | 0 | 0 | 0 | 0 | 0 | 0 | 0 | 0 | 0 | 0 | 0 | 0 | 0 | 0 |
| Concentration / memory problems | 0 | 0 | 0 | 0 | 0 | 0 | 0 | 0 | 0 | 0 | 0 | 0 | 0 | 0 | 0 | 0 | 0 | 0 | 0 | 0 | 0 | 0 | 0 | 0 | 0 | 0 |
| Pain | 0 | 0 | 0 | 0 | 1 | 0 | 0 | 0 | 0 | 0 | 0 | 0 | 0 | 0 | 0 | 0 | 0 | 0 | 0 | 0 | 0 | 0 | 0 | 0 | 0 | 0 |
| Mood | 0 | 0 | 0 | 0 | 0 | 0 | 0 | 0 | 0 | 0 | 0 | 0 | 0 | 0 | 0 | 0 | 0 | 0 | 0 | 0 | 0 | 0 | 0 | 0 | 0 | 0 |
| Jaundice | 0 | 0 | 0 | 0 | 0 | 0 | 0 | 0 | 0 | 0 | 0 | 0 | 0 | 0 | 0 | 0 | 0 | 0 | 0 | 0 | 0 | 0 | 0 | 0 | 0 | 0 |
| Dysphagia | 0 | 0 | 0 | 0 | 1 | 0 | 0 | 0 | 0 | 0 | 0 | 0 | 0 | 0 | 0 | 0 | 0 | 0 | 0 | 0 | 0 | 0 | 0 | 0 | 0 | 0 |
| Hemoglobinuria | 0 | 0 | 0 | 0 | 0 | 0 | 0 | 0 | 0 | 0 | 0 | 0 | 0 | 0 | 0 | 0 | 0 | 0 | 0 | 0 | 0 | 0 | 0 | 0 | 0 | 0 |
| QLQ-30 | 100 |  |  |  |  |  |  |  |  |  |  |  |  |  |  |  |  |  |  |  |  |  |  |  |  | 100 |

Patient 9, female, PNH

| week | 1 | 2 | 3 | 4 | 5 | 6 | 7 | 8 | 9 | 10 | 11 | 12 | 13 | 14 | 15 | 16 | 17 | 18 | 19 | 20 | 21 | 22 | 23 | 24 | 25 | 26 |
| --- | --- | --- | --- | --- | --- | --- | --- | --- | --- | --- | --- | --- | --- | --- | --- | --- | --- | --- | --- | --- | --- | --- | --- | --- | --- | --- |
| Bleeding | 0 | 0 | 0 | 0 | 0 | 0 | 0 | 0 | 0 | 0 | 0 | 0 | 0 | 0 | 0 | 0 | 0 | 0 | 3 | 0 | 0 | 0 | 0 | 0 | 0 | 0 |
| Fever | 0 | 0 | 0 | 0 | 0 | 0 | 0 | 0 | 0 | 0 | 0 | 0 | 0 | 0 | 0 | 0 | 0 | 0 | 0 | 0 | 0 | 0 | 0 | 0 | 0 | 0 |
| Dyspnoea | 0 | 0 | 0 | 0 | 0 | 0 | 0 | 0 | 0 | 0 | 0 | 0 | 0 | 0 | 0 | 0 | 0 | 0 | 0 | 0 | 0 | 0 | 0 | 0 | 0 | 0 |
| Fatigue | 3 | 3 | 3 | 3 | 3 | 3 | 2 | 3 | 3 | 3 | 3 | 2 | 3 | 3 | 2 | 1 | 2 | 1 | 1 | 2 | 3 | 3 | 3 | 2 | 3 | 2 |
| Concentration / memory problems | 2 | 2 | 2 | 2 | 2 | 1 | 1 | 2 | 3 | 2 | 1 | 1 | 1 | 1 | 0 | 0 | 0 | 0 | 0 | 1 | 1 | 1 | 2 | 1 | 1 | 1 |
| Pain | 0 | 0 | 2 | 2 | 2 | 3 | 0 | 3 | 0 | 3 | 0 | 0 | 0 | 0 | 0 | 0 | 0 | 0 | 0 | 0 | 0 | 0 | 0 | 0 | 0 | 0 |
| Mood | 0 | 0 | 0 | 0 | 0 | 0 | 0 | 0 | 0 | 0 | 0 | 0 | 0 | 0 | 0 | 0 | 0 | 0 | 0 | 0 | 0 | 0 | 0 | 0 | 0 | 0 |
| Jaundice | 0 | 0 | 0 | 0 | 0 | 0 | 0 | 0 | 0 | 0 | 0 | 0 | 0 | 0 | 0 | 0 | 0 | 0 | 0 | 0 | 0 | 0 | 0 | 0 | 0 | 0 |
| Dysphagia | 0 | 0 | 0 | 0 | 0 | 0 | 0 | 0 | 0 | 0 | 0 | 0 | 0 | 0 | 0 | 0 | 0 | 0 | 0 | 0 | 0 | 0 | 0 | 0 | 0 | 0 |
| Hemoglobinuria | 0 | 0 | 0 | 0 | 0 | 0 | 0 | 0 | 0 | 0 | 0 | 0 | 0 | 0 | 0 | 0 | 0 | 0 | 0 | 0 | 0 | 0 | 0 | 0 | 0 | 0 |
| QLQ-30 | 67 |  |  |  |  |  |  |  |  |  |  |  |  |  |  |  |  |  |  |  |  |  |  |  |  | 67 |

0 = no symptoms, 1 = mild symptoms, 2 = moderate symptoms, 3 = severe symptoms

**Table 7: Patient interviews**

|  | **Week 3 (n = 9)** | **Month 3 (n = 8)** | **Month 6 (n = 9)** |
| --- | --- | --- | --- |
| Have technical problems occurred? | No = 9 (100%) | No = 8 (100%) | No = 9 (100%) |
| Was the questionnaire easy to understand? | Very easy = 7 (78%)  Fairly easy = 2 (22%) | Very easy = 8 (100%) | Very easy = 8 (89%)  Fairly easy = 1 (11%) |
| How would you rate time consumption for the application? | Pleasant = 9 (100%) | Pleasant = 8 (100%) | Pleasant = 9 (100%) |
| Do you see the application as a burden? | No = 9 (100%) | No = 7 (88%)  A little = 1 (13%) | No = 8 (89%)  A little = 1 (11%) |
| Would you prefer to enter symptoms more/less often? | Less often = 1 (11%)  Same = 7 (78%)  More often = 1 (11%) | Less often = 2 (25%)  Same = 6 (75%) | Less often = 2 (22%)  Same = 6 (67%)  More often = 1 (11%) |
| Have the self-help instructions helped you? |  | No = 2 (25%)  Not much = 4 (50%)  Fairly = 1 (13%)  No answer = 1 (13%) | Not much = 4 (44%)  Fairly = 2 (22%)  Very = 1 (11%)  No answer = 2 (22%) |
| Are the warnings consistent with your symptom experience? |  | Sometimes = 2 (25%)  Often = 2 (25%)  No warnings = 4 (50%) | Sometimes = 3 (33%)  Often = 1 (11%)  Always = 1 (11%)  No warnings = 4 (50%) |
| What device did you use? (multiple answers possible) |  |  | Smartphone = 7  Tablet = 1  Computer = 3 |
| How would you rate the applications usability? |  |  | Easy to use = 9 (100%) |
| What factors have let to lack of compliance? (multiple answers possible) |  |  | Busy = 4  Forgetting = 4  No symptoms = 1 |
| Have you been contacted by your physician? |  |  | No = 9 (100%) |
| Did you see a personal benefit from the application? |  |  | Yes = 5 (56%)  No = 4 (44%) |
| How difficult was the applications integration into daily life? |  |  | Very easy = 9 (100%) |
| Are you satisfied with the application? |  |  | Yes = 9 (100%) |
| Would you continue to use the application? |  |  | Yes = 7 (78%)  No = 2 (22%) |
| Would you recommend the application to other patients? |  |  | Yes = 9 (100%) |

**Table 8: Patient comments**

|  | **Did you have symptoms, which were not mentioned in the questionnaire?** | **Which functions would you like to include into the application?** | **What would you change within the application?** | **Other comments** |
| --- | --- | --- | --- | --- |
| Pt 1 | “Nausea and chest pain.“ | “Reminders for medication and hospital visits would be useful.” | “Graphs for vital parameters and blood values could be improved.” | “Compliance would be higher, if physicians would use the application.”  “Fatigue warning was triggered too early, I wouldn’t see a doctor because of this.” |
| Pt 2 |  |  | “Improve the visual attractiveness.” | “Very interesting application. It’s assuring to have something at hand if symptoms would occur.” |
| Pt 3 |  | “A calendar function with doctors’ visits.” |  | “The application would have been very useful in the beginning of my disease.” |
| Pt 4 |  | “A possibility to ask questions to the caretakers without the need of a personal consultation.” |  | “Advice for self-care is good, although I already know these measures and therefore don’t need them anymore.” |
| Pt 5 |  | “Integration of blood values and vital parameter from the hospital IT system would give a good overview for patients. Additionally the integration of therapies would help to see therapy effect.” |  | “After stem cell transplantation, the symptoms change compared to AA.”  “Self-care advice did not help much, because I have seven years of experience and was monitored closely. It would certainly help others with less experience.” |
| Pt 6 |  |  |  | “Warnings were triggered too early for me.”  “The application is easy to use and would certainly be a help for newly diagnosed patients.”  “I didn’t benefit from the application, because I’m experienced and familiar with my symptoms and management.” |
| Pt 7 |  | “A possibility to exchange experiences with other patients.” | “Design could be more attractive.” | “The application would be a big help in the early stage of the disease. Later the benefit declines.”  “Advice and warnings are helpful when coping with the disease.” |
| Pt 8 |  |  |  | “The application helps with self-reflexion and gives good feedback.” |
| Pt 9 |  |  |  | “For fatigue the questionnaire would be best every three days.” |

**Table 9: Caretaker interviews**

|  | Physician 1 | Physician 2 | Physician 3 | Physician 4 | Physician 5 | Nurse 1 | Nurse 2 |
| --- | --- | --- | --- | --- | --- | --- | --- |
| Have technical problems occurred? | No | Yes, a password reset fail. | No |  |  | Yes, two password reset fails | No |
| Did you have a benefit from the tool? | Unsure | Not much | No | “Yes, because Symptoms are not reported the same as during visits.” | “Yes, for better understanding the patients’ situation.” | “Yes, the questionnaire is an extension of usually asked symptoms in patient visits.” | “Yes, as an overview and for symptom monitoring.” |
| (Physicians) Have you received symptom alerts? | No | Yes | Yes | Yes | “Yes, often. Uncertainty on how to respond was an issue in the beginning.” |  |  |
| (Physicians) Did you react to symptom alerts? |  | No | No | “No, but they were mentioned during visits.” | “No, symptoms were discussed during visits.” |  |  |
| (Nurses) Do you feel confident to manage the symptom reports? |  |  |  |  |  | “Yes, for severe symptoms certainly. Guidelines would be helpful though.” | “Yes, but guidelines would be needed for implementation into routine care.” |
| How do you rate the time effort for the tool? | “Barely any effort.” | “Very little effort.” | “Minimal effort.” | “Small.” |  | “Minimal effort. A time frame should be planned for clinical management.” | “Small, though we were not fully integrated into the workflow.” |
| Are you satisfied with the application? | “Depends on the goal. It doesn’t help physicians a lot, but opens a new possibility for communication between nurses and patients.” |  | “Yes, although the app wasn’t part of treatment and not used much. But it’s good for gathering patient-specific data and symptom overview.” |  |  | “Yes. The tool works flawless and is an interesting extension, especially because patients tend to make a better impression during routine visits.” | “Yes. Especially the symptom feedback for patients my be useful.” |
| Would you continue to use the application? | “Yes, but for several patients we need a time-efficient management.” |  | “Yes, but preferable within the hospital IT system.” |  | “Yes, we need tools in the future to better assist the patients.” | Yes | Yes |
| Would you recommend the tool to others? | “For young doctors certainly, it could help them to gain experience for rare diseases.” |  | Yes |  | “Yes, when patient communication can be improved with it.” | Yes | “Yes, if it can be integrated into daily routine.” |
| Which functions does the tool lack? |  |  | “An alarm function for patients.” | “Therapies should be integrated to for comparison between strategies and better detection of improvements or worsening.” | “A contact function. But it must be certain that this function is monitored closely and leads to an adequate response from clinicians.” | “Functions are good, important is to use the given information for tailored improvements in therapy.” | “A reminder for medication intake and hospital visits would be useful.” |
| Would you improve something within the tool? | “Questionnaire intervals should be adapted to the patients’ needs.” | “The integration into the hospital IT system is important for the workflow.” | “For integration into therapy, the tool needs to be integrated into workflow and managed in real-time. | “For implementation into practice, it is important to have responsibilities and tasks well defined.” | “For implementation into practice we would need to specify tasks between nurses and physicians and train both in the application usage.” | “The tool management must be discussed between nurses and physicians and tasks defined.” | “The cut-offs for symptom alerts must be clear to the caretakers to ensure proper actions.” |
| Comments |  |  |  |  | “It’s important to integrate technical development into patient care. Especially for older, immobile patients this could have a positive impact.” | “The tool could be an enhancement for the care setting without much effort needed.” | “The integration into the hospital IT system and into treatment routine is important for such a tool.” |
